# Assessing the efficacy of various irrigation solutions in dissolving organic tissue

**DOI:** 10.1038/s41598-024-64904-w

**Published:** 2024-06-15

**Authors:** Asu Çakır, Tuğçe Nur Şahin, Özlem Kahveci

**Affiliations:** 1 Pediatric Dentistry Department, Ahmet Kelesoglu Dentistry Faculty, Karamanoglu Mehmetbey University, Karaman, Turkey; 2 Pendik Oral and Dental Health Center, İstanbul, Turkey

**Keywords:** Boric acid, Irritrol, Organic tissue dissolution, Root canal treatment, Sodium hypochlorite, Health care, Medical research

## Abstract

For successful root canal treatment, adequate chemomechanical instrumentation to eliminate microorganisms and pulp tissue is crucial. This study aims to assess the organic tissue dissolving activity of various irrigation solutions on bovine tooth pulp tissue. 40 extracted bovine mandibular anterior teeth (n = 10) were used for the study. Bovine pulp pieces (25 ± 5 mg) were placed in 1.5 ml Eppendorf tubes. Each tooth pulp sample was then covered with 1.5 ml of different irrigation solutions, dividing them into four groups: Group 1 with freshly prepared 5% Boric acid, Group 2 with 5% NaOCl, Group 3 with Irritrol, and Group 4 with Saline. Samples were left at room temperature for 30 min, then dried and reweighed. The efficacy of tissue dissolution ranked from highest to lowest was found to be NaOCl, Boric Acid, Irritrol, and saline (*p* < 0.05). It was observed that the decrease in the NaOCl group was greater than the decrease in the Irritrol and saline groups, and the decrease in the Boric acid group was significantly greater than the decrease in the saline group (*p* < 0.05). It also emphasizes the need for future studies to further investigate the effects of Irritrol and Boric Acid on tissue dissolution.

## Introduction

In dentistry, the success of root canal treatment depends not only on well-shaped canals but also on the elimination of necrotic/vital pulp tissues and microorganisms within the canal. During the shaping of root canals, the use of irrigation solutions facilitates the removal of debris exposed by canal files, leveraging their antimicrobial activity and tissue dissolving effects^1,2^. Moreover, due to the complex anatomical structure of the root canal system, it has been reported that complete preparation cannot be achieved by mechanical preparation alone^3^. In this context, the use and efficacy of root canal irrigations become crucial.

For successful root canal treatment, adequate chemomechanical instrumentation to eliminate microorganisms and pulp tissue is crucial. During canal shaping with files, the use of irrigation solutions facilitates the removal of debris, leverages antimicrobial activity, and utilizes tissue-dissolving effects^1,2^. Additionally, due to the complex anatomy of the root canal system, it's acknowledged that mechanical preparation alone cannot achieve complete preparation^3^ underlining the importance of irrigation solutions in endodontic treatment.

Among all irrigation solutions, NaOCl is often chosen due to its encompassing desirable properties, notably its effectiveness in removing pulp tissue remnants and the organic component of the smear layer generated during mechanical preparation, alongside its antimicrobial efficacy^4^. The tissue-dissolving property of NaOCl can vary depending on its concentration, volume, temperature, pH, and contact time^2,5^. When NaOCl alone is insufficient for smear layer removal, the use of chelating agents like EDTA is recommended^4^.

Recently, boric acid, used as an irrigation agent, has been shown to possess antiseptic, antibacterial, and antifungal properties^6,7^, including significant anti-inflammatory and antimicrobial effects^8–10^. Although its effects on smear layer and dentin mineral content have been explored^11,12^, studies on its tissue-dissolving capabilities are lacking.

Irritrol, a combination of EDTA, chlorhexidine gluconate (CHX), and surfactants, has proven effective in removing the smear layer^13,14^, touted by its manufacturer for quick and effective root canal disinfection. Dissolution of pulp tissue is a sought-after property in endodontic irrigation solutions.

Despite slight differences, bovine pulp tissue is considered more comparable to human pulp than other animal models and histologically similar to human pulp^15,16^, having been used in previous tissue dissolution studies^2,17^.

The efficacy of endodontic treatment relies on several factors, including adequate mechanical instrumentation, irrigation, debridement, and disinfection of the root canal system^1^. It's known that mechanical preparation alone can leave 40–50% of canal walls untouched, potentially creating an environment for residual living tissue fragments where microorganisms can thrive and lead to persistent infections^18–20^. It is stated that NaOCl is the gold standard irrigation agent used in endodontics, and the use of Boric acid or Irritrol cannot replace the use of NaOCl in endodontics. Additionally, Irritrol has been shown to have poor smear layer removal and antibacterial properties^21^. However, literature review reveals limited studies on the tissue-dissolving capabilities of irrigation solutions other than NaOCl, such as Boric Acid and Irritrol. This study aims to evaluate the organic tissue dissolving activity of various irrigation solutions on bovine tooth pulp tissue. The null hypothesis posits that, aside from saline, the other tissue dissolvers used in this study effectively dissolve bovine pulp.

## Methods

### Data acquisition

Our research utilized 40 extracted bovine mandibular anterior teeth (n = 10), obtained from Panagro Meat and Dairy Integrated Facilities (Meram, Konya, TURKEY). Teeth harvested one day before the experiment were stored at − 20 degrees Celsius in a freezer. Before starting the study, the teeth were thawed to room temperature. Using a diamond bur, the teeth were horizontally cut at the enamel-cement junction to separate the crown and root portions. Pulp tissue was extracted from the root canal with fine-tipped forceps, with excess blood and debris washed away with distilled water. The samples were kept in the tubes at room temperature for half an hour, then dried with paper towels and weighed on a precision scale. Then, the pulp was divided into pieces weighing approximately 25 ± 5 mg with the help of a #12 scalpel. These weighed bovine pulp segments were placed into 1.5 ml Eppendorf tubes. Each tooth pulp sample was then covered with 1.5 ml of different irrigation solutions, dividing them into four groups: Group 1 with freshly prepared 5% Boric acid, Group 2 with 5% NaOCl (pH ~ 12) (Microvem, Istanbul, Turkey), Group 3 with Irritrol (Essential Dental Systems, New Jersey, USA), and Group 4 with Saline (Polifleks, Polifarma, İstanbul, Turkey). The samples were dried again and weighed again on a precision balance. The difference between the initial and final measurements was statistically analyzed.

### Statistical analysis

In the G*Power 3.1.9.7 power analysis performed with the parameters of 80% power, 0.05 alpha error rate, and 0.58 effect size, it was calculated that we needed 40 samples in total.

In this study, data were analyzed using the licensed IBM SPSS 21 (SPSS Inc., Chicago, IL, ABD) software package. To determine whether variables followed a normal distribution, the Shapiro–Wilk and/or Kolmogorov–Smirnov tests were utilized due to the sample sizes. A significance level of 0.05 was adopted for interpretations; variables were considered not to follow a normal distribution if *p* < 0.05, and to follow a normal distribution if *p* > 0.05. In examining differences between groups, the Kruskal–Wallis H test was employed for variables not following a normal distribution. Significant differences identified by the Kruskal–Wallis H test led to further analysis with the Post-Hoc Multiple Comparison Test to pinpoint the groups between which differences existed. For examining differences between two related variables that did not follow a normal distribution, the Wilcoxon Test was used. A significance level of 0.05 was applied for interpretations, where *p* < 0.05 indicated a significant difference, and *p* > 0.05 indicated no significant difference.

The initial measurement values of the samples before any procedure show no statistically significant difference between groups as seen in Table 1 (*p* > 0.05).

**Table 1:** Differences between groups in terms of ınitial measurement values.

		Group						Kruskal Wallis H test		
		n	Mean	Median	Min	Max	SD	Mean rank	H	p
Initial	Boric acid	10	25.25	24.75	22	29.5	2.51	22.85	7.345	0.062
	NaOCl	10	25.38	25.75	20.8	29.5	2.77	24.9		
	İrritrol	10	22.69	22.65	20	24.5	1.65	12		
	Saline	10	24.83	24.55	20.1	29.8	3.16	22.25		
	Total	40	24.54	24.3	20	29.8	2.71			

As presented in Table 2, there is a statistically significant difference between the groups in terms of the final measurement values after the samples were exposed to the solutions (*p* < 0.05). The final measurement value of the NaOCl group is significantly lower compared to the Irritrol and saline groups; similarly, the final measurement value of the Boric Acid group is significantly lower compared to the saline group.

**Table 2:** Differences between groups in terms of final measurement values.

		Group						Kruskal Wallis H test		
		n	Mean	Median	Min	Max	SD	Mean Rank	H	p
Final	Boric acid	10	18.21	18.8	11.9	23.2	3.78	17	27.584	0.001
	NaOCl	10	12.96	12.1	9.7	17.4	2.9	6.8		
	İrritrol	10	21.34	21.7	19.2	22.4	1.21	25.3		
	Saline	10	24.81	24.5	20.1	29.7	3.14	32.9		
	Total	40	19.33	20.7	9.7	29.7	5.23	2–3 2–4 1–4		

Statistically significant differences between groups in terms of change values are observed in Table 3 (*p* < 0.05). The decrease in the NaOCl group is significantly greater compared to the decreases in the Irritrol and saline groups; similarly, the decrease in the Boric acid group is significantly greater than the decrease in the saline group. It was observed that the tissue dissolving properties of the NaOCl group were statistically significantly higher than the other groups.

**Table 3:** Differences between groups in terms of change values.

		Differences						Kruskal Wallis H test		
		n	Mean	Median	Min	Max	SD	Mean rank	H	p
Group	Boric acid	10	− 7.04	− 7.3	− 10.3	− 3.1	2.68	14.15	35.18	0.001
	NaOCl	10	− 12.42	− 12.65	− 19.5	− 6	4.07	6.85		
	İrritrol	10	− 1.35	− 1.15	− 2.2	− 0.8	0.57	25.5		
	Saline	10	− 0.02	0	− 0.1	0	0.04	35.5		
	Total	40	− 5.21	− 2.65	− 19.5	0	5.52	2–3 2–4 1–4		

### Ethics approval

This study was conducted in accordance with Arrive guidelines and ethical approval was received from Karamanoğlu Mehmetbey University Faculty of Medicine Local Scientific Medical Research Ethics Committee (01–2024/21).

## Discussion

Considering that any remaining pulp in the root canal may be linked to treatment failure, the dissolution rate of pulp tissue in irrigation solutions should be considered. Postoperative pain is more common in vital pulpectomies than in non-vital cases, possibly due to residual pulp tissue in the root canal^22^. With this in mind, this study investigated the tissue dissolving effect of Boric Acid and Irritrol in addition to the commonly preferred NaOCl, to provide a new perspective for clinical applications. The results are consistent with literature that indicates NaOCl's tissue dissolving capabilities^17,23–25^, showing statistically significant higher efficacy compared to the other three irrigation solutions used in the study. However, there have been no studies found on the tissue dissolving effects of Boric Acid and Irritrol. In this current study, Boric Acid's tissue dissolution was significantly higher than Irritrol and saline, but lower than NaOCl. Moreover, the irritant nature of NaOCl, its unpleasant taste and odor, are significant drawbacks^26–29^. A study indicated that the use of rubber dams during primary tooth canal treatment in our country is at quite low levels^30^, suggesting clinicians should lean towards more tolerable materials. On the other hand, Boric Acid is known to be odorless and tasteless and can be added to mouthwash, and it is known to increase the mineral density of bone^31,32^.

Haapasalo et al.^33^ emphasized in their study that an ideal irrigation solution should dissolve both organic and inorganic tissue remnants. With this perspective, we consider both Boric Acid and Irritrol could be preferred as irrigation solutions. Although the tissue dissolving effects of these two solutions are not yet fully known, existing studies claim they have the positive effects necessary for use in canal irrigation^6–14^. However, in this study, Irritrol ranked behind NaOCl and Boric Acid in tissue dissolving efficacy but was more effective than saline.

The use of Eppendorf tubes, allowing for a larger volume of irrigation solution in contact with the pulp tissue and more surface area contact, may facilitate greater tissue dissolution. When compared to clinical conditions, this can be considered a limitation of the study.

One of the main drawbacks of this study is the model used for evaluating tissue dissolution. A tooth model should have been used to simulate the clinical scenario (Int Endod J 2022;55:295–329). Dipping the tissue samples directly into the test agents in Eppendorf tubes might overestimate the results.The results of this study support the null hypothesis that all solutions, except for saline, are effective as tissue dissolvers, with NaOCl showing significantly greater efficacy than all other solutions used.

## Conclusion

This study has shown that sodium NaOCl is a more effective tissue dissolver than the other solutions used. It also emphasizes the need for future studies to further investigate the effects of Irritrol and Boric Acid on tissue dissolution.

## Data Availability

Data is provided within the manuscript and in supplementary information files.
